# Supplementary material for: The Association between Muscle Quantity and Overall Survival Depends on Muscle Radiodensity: A Cohort Study in Non-Small-Cell Lung Cancer Patients
Source: J Pers Med. 2022 Jul 21;12(7):1191. doi: 10.3390/jpm12071191 (PMC9322608; doi:10.3390/jpm12071191)
Supplement: Supplementary file 1 [file jpm-12-01191-s001.zip › jpm-1792656-supplementary.pdf]

Journal of Personalized Medicine

Supplementary Materials

The Association between Muscle Quantity and Overall Survival Depends on Muscle Radiodensity: A Cohort Study in Non-Small-Cell Lung Cancer Patients

Appendix for: "The association between muscle quantity and overall survival depends on muscle radiodensity: a cohort study in non-small cell lung cancer patients"

Sample size simulations

We used simulations to calculate the required sample size. Performing these simulations requires assumptions on three things: the outcome model (i.e. hazard ratios and baseline hazard function), the correlation between predictor variables and the censoring distribution.

As Sjøblom et al. (1) present the most complete multivariable analysis, we use their results (presented in their Table 3) as the basis for assumed parameter values. The assumed parameter values are presented in Table 1. The assumed parameter value for our target parameter SMISMD (the linear interaction between skeletal muscle index, SMI, and skeletal muscle radiodensity, SMD) was taken to be halfway between the hazard ratio for SMI and the hazard ratio for SMD on the log-hazard ratio scale. As the interpretation of the absolute value of a hazard ratio relies on the scale of the variable, the hazard ratio for the interaction term was rescaled by multiplying with the standard deviation of SMD and dividing by the standard deviation of SMISMD in each simulated dataset. The hazard ratios for histology subtypes were not given so we assumed values for these.

Table S1. Hazard ratios for sample size calculations. Adenocarcinoma is the reference category for histology group. BMI: body mass index, PS: ECOG performance score (0 is the reference category), SMD: skeletal muscle radiodensity, SMI: skeletal muscle index, SMISMD: interaction term between SMI and SMD.

Term	hazard_ratio	log_hazard_ratio
Age	0.99	-0.010
Male sex	0.77	-0.261
Histology: other	1.22	0.2
Histology: squamous	1.35	0.3
BMI	0.99	-0.01
PS 1	1.24	0.215
PS >=2	1.89	0.636
SMD	0.98	-0.017
SMI	0.99	-0.010
SMISMD	0.99	-0.014

For all variables, marginal statistics (mean and standard deviations for continuous variables, frequency tables for discrete variables) were extracted from (1). As a frequency table for the four NSCLC stages was not available from (1), we used two additional

publications to reconstruct the frequency table for clinical stage. Dolan et al. provided the relative frequencies of stages I, II and III (2). Abbass et al. provided relative frequencies for stages III and IV (3). These relative frequencies were used to reconstruct a single full frequency table for all four stages. In addition to the hazard ratios for the individual parameters, the power also depends on the correlation between the predictor variables. As a complete covariance matrix for all variables was not available, we simulated covariate data using covariance structures induced by different Clayton copulas (4). Copulas are multivariate cumulative distribution functions whose marginal distributions are uniform on the unit interval. A Clayton copula can be defined using the known marginal statistics of the observed variables and a single unknown correlation parameter. This can be done by translating the marginal distributions (assumed to be Gaussian for continuous variables, binomial for binary variables and discretized Gaussian for discrete variables) of the variables to cumulative distribution functions. The inverse of these cumulative distribution functions are also uniform on the unit interval by definition and can then be identified with the marginal distributions from the copula. The relationship between the Clayton copula parameter and the average Pearson correlation coefficient of variables generated from such a copula is presented in Table 2.

Table S2. Clayton copula parameter versus average Pearson correlation coefficient of two variables simulated by a Clayton copula with that parameter value.

Copula parameter	*Pearson correlation*
0.1	0.043
0.2	0.115
0.3	0.231
0.4	0.258
0.5	0.326
0.6	0.398
0.7	0.386
0.8	0.408
0.9	0.486
1	0.510

Finally, the power also depends on the marginal survival distributions and the censoring distributions. We estimated the marginal survival distributions per stage, and the censoring distribution for all stages from our data. We used the parametric power generalized Weibull model (5) to estimate these survival distributions and to simulate survival times. To prevent extreme outliers with high leverage, simulated survival times over 15 years were censored. Kaplan-Meier estimates and power generalized Weibull estimates of the marginal survival distributions per stage are presented in Figure 1.

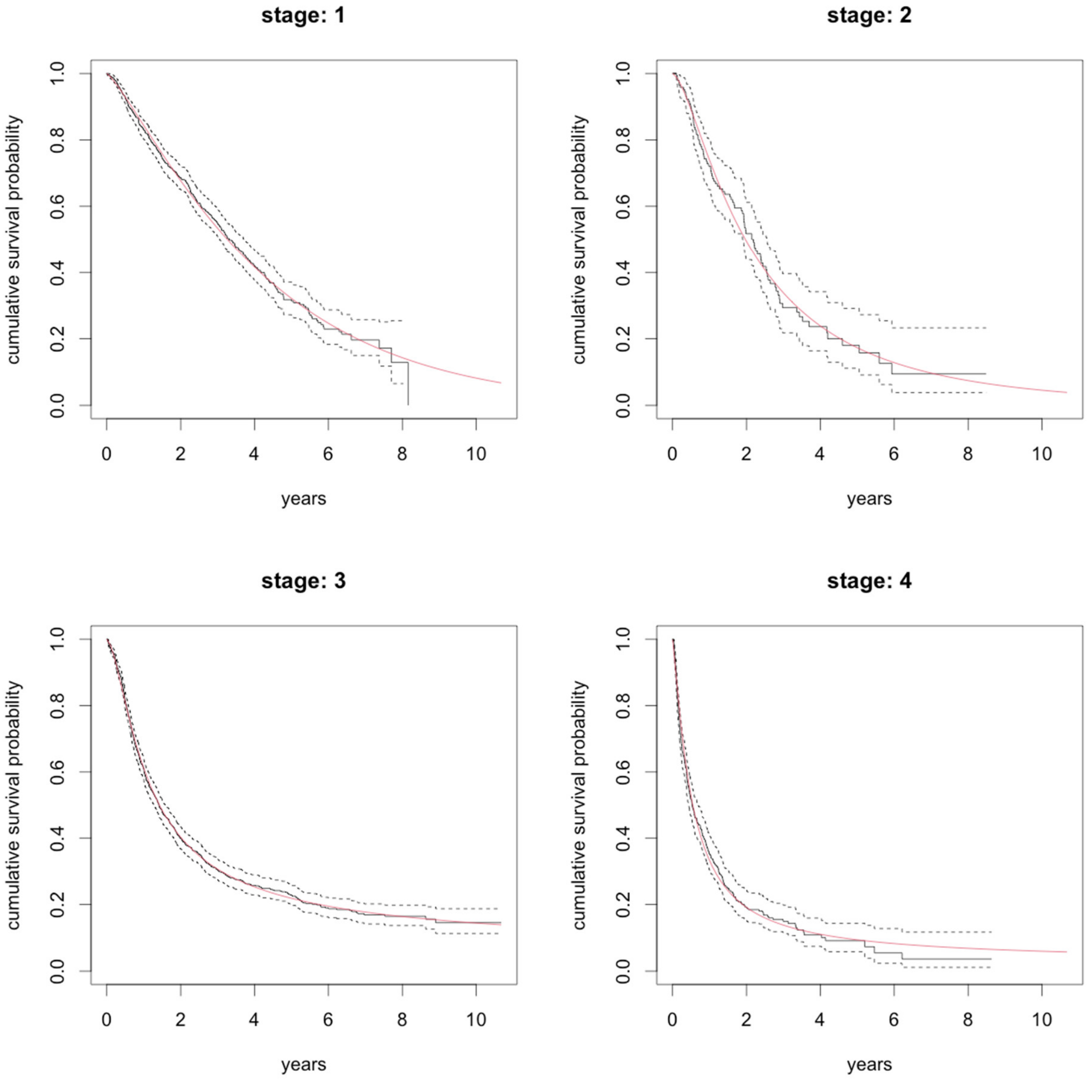

Figure S1. Marginal survival distributions per stage. The Kaplan-Meier estimate is presented with the solid black line, accompanied by a 95% confidence interval indicated with the dotted black line. The parametric power generalized Weibull estimate that was used in the simulations is indicated with the red line.

We calculated the power to detect the pre-specified interaction hazard ratio at a 0.05 significance level using a two-sided Student's T-test. We calculated the power over the following grid of values: Copula parameter 0.1, 0.25, 1.0; sample size 500, 1000, 2000. For each of the 9 combinations we simulated 1000 datasets. The power was defined as the number of times a significant result was detected divided by the total number of simulations for that setting. The results of the power analysis are presented in Table 3.

Table S3. Results of power analysis for the interaction term between skeletal muscle index (SMI) and skeletal muscle radiodensity (SMD).

Power	*Sample size*	*Copula parameter*
0.576	500	0.1
0.551	500	0.25
0.517	500	1
0.887	1000	0.1
0.865	1000	0.25
0.791	1000	1
0.99	2000	0.1
0.992	2000	0.25
0.968	2000	1

Table S4. Overview of different target regions for radiotherapy per stage.

target	missing	stage I	stage II	stage III	stage IV
**missing**	0	0	0	0	1
**brain**	1	0	0	0	7
**hilus**	14	0	7	11	5
**hilus, supraclavicular**	1	0	0	0	1
**lung**	456	667	92	179	126
**lung, hilus**	19	3	7	9	3
**lung, mediastinum**	158	26	28	571	121
**lung, mediastinum, hilus**	3	0	0	16	1
**lung, mediastinum, supraclavicular**	5	0	0	8	2
**lung, supraclavicular**	3	0	0	3	2
**lung, thoraxwall**	4	0	2	2	7
**lung, thoraxwall, vertebra**	1	0	0	0	0
**lung, vertebra**	2	0	0	1	0
**mediastinum**	30	5	0	43	19
**mediastinum, hilus**	13	0	0	5	4
**mediastinum, hilus, thoraxwall**	0	0	0	1	0
**mediastinum, hilus, vertebra**	0	0	0	0	1
**mediastinum, supraclavicular**	0	0	0	1	2
**mediastinum, vertebra**	0	0	0	0	1
**other**	43	12	7	15	28
**plexus**	0	0	0	0	1
**supraclavicular**	2	0	0	0	1
**thoraxwall**	8	1	2	4	8
**thoraxwall, vertebra**	1	0	0	1	1
**vertebra**	3	0	0	1	1

Table of parameter estimates

Table S5. Estimates of all parameters in the full model with linear interaction term and without stratification of the interaction. The estimates are provided on the log-hazard ratio scale. All continuous variables are scaled to unit variance. Higher order cubic spline terms of continuous variables are not scaled to unit variance which explains the otherwise extremely high parameter estimates. ECOG performance score 0 is the reference category for ecog_bin1 and ecog_bin2. Histology type adenocarcinoma is the reference category for the other histology types.

term	estimate	std.error	statistic	df	p.value
**age**	-0.018	0.122	-0.144	1329.330	0.886
**sex_maleTRUE**	0.188	0.097	1.929	713.392	0.054
**histono_pa**	0.002	0.127	0.015	316.727	0.988
**histoother**	0.194	0.106	1.831	1525.555	0.067
**histosquamous**	0.208	0.089	2.336	1524.444	0.020
**ecog_bin1**	0.099	0.115	0.857	338.536	0.392
**ecog_bin2**	0.511	0.113	4.515	381.416	0.000
**bmi**	0.043	0.216	0.198	325.775	0.843
**smi**	-0.243	0.184	-1.315	345.824	0.189
**smd**	0.053	0.197	0.268	501.070	0.789
**age1**	0.183	0.535	0.342	1339.720	0.733
**age2**	0.828	3.376	0.245	1349.396	0.806
**bmi1**	-1.802	1.685	-1.069	439.175	0.286
**bmi2**	12.337	8.705	1.417	485.333	0.157
**smi1**	1.127	1.323	0.852	405.681	0.395
**smi2**	-2.792	6.181	-0.452	413.667	0.652
**smd1**	-0.712	0.713	-0.998	509.379	0.319
**smd2**	5.390	5.772	0.934	537.145	0.351
**age3**	-3.845	7.203	-0.534	1345.819	0.594
**bmi3**	-18.958	11.953	-1.586	514.767	0.113
**smi3**	0.486	8.643	0.056	409.716	0.955
**smd3**	-7.733	11.220	-0.689	554.587	0.491
**smismd**	-0.089	0.031	-2.868	391.247	0.004
**age:c_stage_earlyTRUE**	-0.160	0.288	-0.556	463.172	0.579
**sex_maleTRUE:c_stage_earlyTRUE**	0.031	0.142	0.218	709.628	0.828
**histono_pa:c_stage_earlyTRUE**	0.006	0.200	0.033	538.576	0.974
**histoother:c_stage_earlyTRUE**	-0.008	0.242	-0.032	1228.706	0.974
**histosquamous:c_stage_earlyTRUE**	0.184	0.196	0.939	1240.738	0.348
**ecog_bin1:c_stage_earlyTRUE**	-0.506	0.190	-2.659	281.168	0.008
**ecog_bin2:c_stage_earlyTRUE**	-0.417	0.185	-2.257	326.862	0.025
**bmi:c_stage_earlyTRUE**	-0.089	0.337	-0.264	244.152	0.792
**smi:c_stage_earlyTRUE**	-0.017	0.292	-0.059	267.759	0.953
**smd:c_stage_earlyTRUE**	-0.171	0.222	-0.770	611.886	0.441
**age1:c_stage_earlyTRUE**	0.498	1.004	0.496	762.491	0.620
**age2:c_stage_earlyTRUE**	-3.642	5.699	-0.639	902.736	0.523
**bmi1:c_stage_earlyTRUE**	-0.798	2.690	-0.297	335.899	0.767
**bmi2:c_stage_earlyTRUE**	3.673	14.236	0.258	361.337	0.797
**smi1:c_stage_earlyTRUE**	1.101	2.166	0.508	299.204	0.612
**smi2:c_stage_earlyTRUE**	-6.375	10.000	-0.638	320.489	0.524
**smd1:c_stage_earlyTRUE**	0.162	0.949	0.171	480.186	0.865
**smd2:c_stage_earlyTRUE**	2.547	8.788	0.290	422.612	0.772

**age3:c_stage_earlyTRUE**	7.489	11.143	0.672	1000.686	0.502
**bmi3:c_stage_earlyTRUE**	-3.758	19.964	-0.188	372.093	0.851
**smi3:c_stage_earlyTRUE**	9.752	13.834	0.705	333.289	0.481
**smd3:c_stage_earlyTRUE**	-9.027	18.269	-0.494	406.953	0.621

References

1. Sjøblom B, Grønberg BH, Wentzel-Larsen T, Baracos VE, Hjermstad MJ, Aass N, et al. Skeletal muscle radiodensity is prognostic for survival in patients with advanced non-small cell lung cancer. Clinical Nutrition. 2016;35(6):1386–93.
2. Dolan RD, Maclay JD, Abbass T, Colville D, Buali F, MacLeod N, et al. The relationship between 18F-FDG-PETCT-derived tumour metabolic activity, nutritional risk, body composition, systemic inflammation and survival in patients with lung cancer. Scientific Reports [Internet]. 2020;10(1). Available from: https://www.scopus.com/inward/record.uri?eid=2-s2.0-85096909045&doi=10.1038%2fs41598-020-77269-7&partnerID=40&md5=dedc04a0cc3d872c6f11ab44a6e4bb0d
3. Abbass T, Dolan RD, MacLeod N, Horgan PG, Laird BJ, McMillan DC. Comparison of the prognostic value of MUST, ECOG-PS, mGPS and CT derived body composition analysis in patients with advanced lung cancer. Clinical Nutrition ESPEN. 2020;40:349–56.
4. Yan J. Enjoy the Joy of Copulas: With a Package copula. Journal of Statistical Software. 2007 Oct 8;21(1):1–21.
5. Burke K, Jones MC, Noufaily A. A Flexible Parametric Modelling Framework for Survival Analysis. Journal of the Royal Statistical Society: Series C (Applied Statistics). 2020 Apr;69(2):429–57.
